# Estimating the potential effects of COVID-19 pandemic on food commodity prices and nutrition security in Nepal

**DOI:** 10.1017/jns.2020.43

**Published:** 2020-10-09

**Authors:** Samrat Singh, Sara Nourozi, Laxman Acharya, Sridhar Thapa

**Affiliations:** 1 School of Public Health, St Mary's Campus, Faculty of Medicine, Imperial College London, Norfolk Place, London W2 1PG, UK; 2 Partnership for Child Development, Faculty of Medicine, Imperial College London, Norfolk Place, London W2 1PG, UK; 3 Partnership for Child Development-HGSF Nepal Project, ANSAB, New Baneshwor, Kathmandu, Nepal; 4 UN World Food Programme, Lalitpur, Nepal

**Keywords:** Nutrition security, COVID-19, Micronutrients, Nepal

## Abstract

The objective of the paper is to analyse changes in food commodity prices and estimate the potential effects of food price change on nutrition security in Nepal in the context of COVID-19 contagion control measures. It presents a comparative intra-country observational study design looking at events before and during the pandemic (after implementation of contagion control measures). The study design includes three districts, enabling comparison between diverse agro-ecological zones and geographical contexts. The methodology consists of primary data collection, modelling and quantitative analysis. The analysis is based on actual school meal food baskets which represent culturally and nutritionally optimised food baskets, developed by the local community and notional typical household food baskets. End May/early June 2020 is the ‘Post-COVID-19’ reference point, the same time period in 2019 i.e. June 2019 is the ‘Pre-COVID-19’ reference point. The study finds a substantial increase in food commodity prices across food groups and districts with marked inter-district variation. For school meal basket, all micronutrients show large average declines ranging from 9⋅5 % for zinc to 11 % for vitamin-A. For household food baskets on average, vitamin-A reduced 37 % followed by iron at 19 %, reduction in zinc is low due to the high zinc content in whole grain cereals. COVID-19 control measures are likely to have contributed to substantial price inflation over the reference period with potentially damaging effects on nutrition security in Nepal with serious implications for vulnerable populations.

## Introduction

The COVID-19 pandemic has caused a global public health crisis on an unprecedented scale, affecting nearly every country and community on the planet. As the disease continues to spread, we are beginning to get an initial glimpse of the destruction it leaves in its wake. The pandemic and contagion control measures have ravaged economies leading to millions of lost jobs and livelihoods. The need to divert community health and clinical resources and social distancing requirement have led to the disruption of public health and safety net programmes. The most widely used contagion control instrument has been lockdowns and curfews, allowing for only very restricted movement of goods and people.

In Nepal, the national lockdown commenced in the last week of March 2020. It included a ban on transport throughout the country and closure of schools, offices and markets. While all sectors of the economy suffer due to such measures, the impact on agriculture and food availability can be particularly debilitating and profound. Countries such as Nepal, which are resource poor with fragile agricultural economies and underdeveloped supply chains are especially vulnerable. The cumulative impact of lost incomes, disrupted food supply, interrupted safety net programmes and reduced coverage of health interventions can lead to a critical food and nutrition crisis and increase in infant and maternal mortality^(1,2)^.

One of the most immediate drivers of food and nutrition insecurity is the increase in food prices. As a coping strategy, poor households replace micronutrient-rich foods such as animal-sourced foods and, fruits and vegetables with cheaper energy-dense foods to preserve caloric intake^(3–5)^. The link between high food prices and food insecurity is well-established^(4,6,7)^. Higher food prices are known to increase malnutrition in young children and mothers especially in low-income and mid-income countries^(7–9)^. The consequences of malnutrition are especially severe for the youngest demography, affecting the development of cognitive abilities which can have significant long term and irreversible impacts on health^(10–12)^. Studies have also shown that children who experience poor nutrition *in utero* and at a very young age suffer from lifelong negative effects in the form of lost human capital, decreased productivity and lower earnings^(13,14)^.

The pandemic and response measures are causing food and nutrition distress for millions of poor households around the world. As case numbers continue to rise and food production is disrupted, the possible final toll on the health and well-being of an entire generation presents a sobering picture. As is always the case, vulnerable populations in low-income countries are likely to suffer the most. In order to better inform policy and catalyse responses, quantification of the potential impact of the pandemic on food and nutrition security in low-income countries is required. Such analysis will also help contribute to further research on the medium and long-term impacts of the pandemic on food security and public health.

The present paper investigates the impact of COVID-19 control measures on food prices and models the effect of food price changes on nutrition quality of diets. It specifically examines the effect of price changes on micronutrient intake. The study design includes three districts, enabling comparison between diverse agro-ecological zones and geographical contexts. The paper applies two different approaches to analyse the potential effect of food price increase on the nutritional quality of diets. School meal food basket (SMFB) provides a useful analytical platform to estimate the most realistic effect of price inflation on cost and diet quality based on local, balanced and diversified weekly diets. However, this does not tell us how the diets in the poorest households of the country will be impacted. In order to understand this aspect, the study constructed typical household food baskets (THFBs) for the poorest quintile (quintile-1) for each district and analysed the cost and diet quality implications of food price changes for these baskets.

The structure of the paper is as follows. Section ‘Methods’ details the approach, methods and materials applied in the study. Methods and materials for each main topic are presented in detail. Section ‘Results’ describes the results, covering cost and diet quality estimates and analysis. Finally, Section ‘Discussion’ summarises and analyses the main results, discusses their implications and highlights some limitations of the study.

## Methods

The present paper presents a comparative intra-country observational study analysing events before and during the pandemic (after implementation of contagion control measures). The design includes primary data collection, modelling and quantitative analysis. The Meal Planning Tool (MPT), a linear programming software developed by Partnership for Child Development (PCD), Imperial College London, was used for nutrient estimations and costings. MPT was coded with relevant food and nutrition data for Nepal.

### Nutrient references

Nutrient data for all commodities is derived from the Nepal Food Composition Table (FCT), 2017, issued by the Department for Food Technology and Quality Control, Government of Nepal. Nepal FCT provided nutrient information for over 567 food items. It does not include zinc values, and occasionally some nutrient values were missing for some commodities and some commodities were missing as such. To address the issue of zinc values and other missing information, data were used from India and Bangladesh FCT's based on the assumption that neighbouring countries would have similar food compositions.

For nutrient requirement estimations, FAO/WHO guidelines and guidelines from India on Estimated Energy Requirement (EER), safe level of intake, acceptable macronutrient distribution range (AMDR), safe level of intake and Recommended Dietary Allowance (RDA), for the specific age group are used, as currently there are no Nepal specific nutrient reference values. Details on reference values for school meals and typical household are provided in the latter part of this section.

### Site selection

We purposively selected three districts of Nepal, representing the main agro-ecological zones and geographical contexts. Bardiya is in *Terai (plains)*, with an open land border with India, Sindhupalchok is in *mid-hills* with a restricted land border with China and Jumla is in the *high mountains*. There are also marked differences in the wealth distribution and food security of the study districts based on the different ecological zones, as can be observed from Table 1.

**Table 1.** Wealth distribution and HH food insecurity of study districts (DHS, 2016)

Ecological zone/District	Wealth quintile – %Age population					HH food insecurity – %Age distribution	
	Q-1	Q-2	Q-3	Q-4	Q-5	Moderate	Severe
Terai/Baridya	5⋅7	19⋅1	28⋅9	26⋅4	19⋅9	28⋅9	13⋅8
Hills/Sindhupalchok	31⋅0	20⋅5	11⋅2	22⋅6	22⋅6	24⋅4	10⋅0
Mountains/Jumla	57⋅8	23⋅4	8⋅3	5⋅7	4⋅9	19⋅1	9⋅2

### Food price data and reference period

The reference period for this analysis is aimed at capturing the potential initial effect of COVID-19 control measures on food prices and diet quality in Nepal. The national lockdown in Nepal due to COVID-19, with a ban on movement of people and goods, commenced on 25 March 2020. In order to allow for some time lag, we chose end May/early June 2020 as the ‘Post-COVID-19’ reference point. To ensure comparability, we chose the same time period in 2019 i.e. June 2019 as the ‘Pre-COVID-19’ reference point.

Phone surveys were undertaken to collect food commodity prices for this period from the three study districts. SMFB provided the list of commodities for price data collection. It consists of a wide range of commodities, around 26 food items on average in each district, from across food groups. Condiments, spices and commodities used in small amounts such as chilly and ginger were not included. Table 2 indicates the number of food commodities by food group for each district for which price data was collected.

**Table 2.** Number of food items in food commodity sample by food group/study district

District	Cereals	Legumes/Pulses	Fruits/vegetables	Roots/Tubers	Animal protein	Total
Baridya	4	6	15	3	3	31
Sindhupalchok	5	5	8	3	3	24
Jumla	7	7	11	2	1	23

Prices were collected from two different independent sources, i.e. traders and farm cooperatives. Price data were verified from a third source, i.e. the World Food Programme (WFP) Nepal's Vulnerability Analysis and Mapping (VAM) unit, by comparing data for some commodities for each district. For the ‘Pre-COVID-19’ reference point, we chose the same time period in 2019, i.e. May/June 2019. The ‘Pre-COVID-19’ prices were collected during school meal design workshops and market surveys. Bardiya and Sindhupalchok base prices were collected in August 2018 and Jumla prices were collected in June 2019. For the purposes of the present study, Bardiya and Sindhupalchok prices were inflation adjusted using inflation data from Nepal Central Bank, till June 2019 and validated with actual June 2019 prices for some commodities.

### Food price changes

The food commodity data were coded separately for five food groups. Changes in prices for each district by food group were then analysed. Prices for 2019 and 2020 were averaged for each food group to calculate the change in prices over our reference period.

### School meals

The SMFBs represent culturally, locally and nutritionally optimised food baskets, developed by the local community and currently served as school meals. All school meals were designed at the district level, in participatory workshops based on menu development guidelines. The meals are based on local taste, culture, production and availability for each district. For each district, six separate meals were designed for each day of the school week. The six meals have been combined to comprise an SMFB for this analysis.

Recommended nutritional intakes for school meals were calculated based on FAO/WHO guidelines as endorsed by *Nepal School Meals Nutrition Technical Working Group*. Table 3 summarises the nutrient reference values for school meals in Nepal.

**Table 3.** Recommended nutrient intake reference values (weekly) for school meals

Nutrient	Recommended amount/week	Measure	Details
Energy (kcal)	11 100	Estimated average requirement (EAR)^(15)^	Boys 7–10 = 2100 kcal Girls 7–10 = 1800 kcal
Fat (kcal)	369⋅6	Acceptable macronutrient distribution range (AMDR)^(16)^	Max 30 % of calories from energy
Protein (g)	177	Safe level of intake^(17)^	Based on India's values, child weight = 25⋅1 kg, Grams protein per kg = 1⋅17 Age = 7–9
Zinc (mg)	67⋅2	Recommended daily allowance (RDA)^(18)^	Low bioavailability, 15 % Age = 7–9 years
Vitamin-A (μg)	3000	Safe level of intake	Age = 7–9 years of age
Iron (mg)	106⋅8	Recommended daily allowance (RDA)^(18)^	Low bioavailability, 5 % Age = 7–10 years
Calcium (mg)	4200	Recommended daily allowance (RDA)^(18)^	Range = 600–800 mg

We calculated the unit cost of school meals (per child/per meal/per day) for the two points in time based on the food price data. As each set for each district consists of six different meals for six days of the week which are repeated every week, our unit of analysis for nutrient supply is one week. Prices for each actual daily meal vary depending on ingredients. The unit cost is, thus, derived from the average cost of the six meals.

In order to estimate the impact of increased prices on the nutrient intake, we undertook meal adjustment modelling for all school meals for the three districts. The objective of this modelling was to understand and estimate the net effect of price changes on nutrient intake. The basic idea was to adjust all daily meals for the three districts, i.e. eighteen meals, based on 2020 food prices, such that the total cost of each meal remains the same as our baseline reference cost in 2019. This required a reduction in the quantity of each ingredient by a certain amount. We considered different methods for meal adjustment. Each meal was designed by the community with fixed proportions of different ingredients as per local culinary practices, and therefore, we considered it important to maintain the correct proportion of ingredients in a meal to preserve its integrity. Finally, the following method was applied which keeps each meal intact in terms of ingredients and proportions. The main computational steps for this method are as follows:

- Calculate the ratio of the total meal cost 2019 (Pre-COVID-19) and 2020 (Post-COVID-19).
- Apply this ratio to each ingredient.
- Apply this ratio to each nutrient value of the 2019 cost of meal.

*Adjusted ingredient weight* = *original weight of ingredient* × (*2019 cost of meal* ÷ *2020 cost of meal*)

After all the eighteen meals were adjusted, the nutrient composition of all the adjusted meals was calculated in terms of the amount of each nutrient and as percentage of the RDA using the MPT. Total nutrient weekly supply was then calculated for each district based on the six district-specific meals. These values (2020) were compared with the base reference values (2019).

### Typical household food basket

THFBs were developed specifically for each district and represent notional diets being consumed by the poorest quintile of the population. Typical food baskets have their limitations in that they provide a restricted model of what households actually consume over a week or a month. While the limitations remain, the present paper has attempted to develop credible THFBs based on multiple primary and secondary evidence.

A THFB was designed for a HH in the first wealth quintile (quintile-1) for each district. Quintile-1 and quintile-2 account for 31 and 22 % of the rural population, respectively (DHS 2016). Our population of interest is quintile-1 and quintile-2 as these segments are most vulnerable to the effects of food price inflation on food and nutrition security. In Nepal, quintile-1 and quintile-2 spend 64⋅2 and 62⋅4 % of the total household budget on food, respectively. We designed THFB for both quintiles, but on further analysis, only quintile-1 THFBs was included. The difference between the two baskets was insubstantial and, therefore, the quintile-2 THFBs were not included in the paper.

#### Method of Household and THFB construction

The construction of the three THFB involved approximations of household composition, household food consumption and expenditure data. The household of six members for quintile-1 was determined based on national survey findings^(19)^. These assumptions related to age and gender were made and validated from the analysis of primary and secondary data sources^(19–22)^. The household profile was kept the same for all nutrient and cost assessments of the THFBs, by district.

Nutrient reference intakes were used to calculate the daily nutritional recommended amounts for each household member. The average energy requirements were based on age, weight, gender and level of physical activity for each household member. Adult household members were assumed to have moderate physical activity levels (PALs), from 1⋅70 to 1⋅90, except for the older female who had sedentary levels of physical activity at 1⋅60. The weight of each family member was estimated using data collected from previous research^(23)^. The energy requirements were based on estimated average requirement references sourced from the Joint FAO/WHO/UNI expert Consultation in 2001^(15)^. Safe level of protein intakes was estimated based on guidelines within the WHO/FAO/UNU Joint Expert Consultation on Protein and Amino Acid Requirements in Human Nutrition^(24)^. The RDA reference values used for zinc, iron, vitamin C and calcium, and the safe level of intake reference values for vitamin-A were based on the FAO/WHO Guidelines on Vitamin and Mineral Requirements in Human Nutrition^(18)^. Low bioavailability reference values for zinc (15 %) and iron (5 %) were used, given that the Nepalese diet contains high levels of antinutrients and low amounts of animal-based foods^(18)^. Fat was limited to contributing up to 30 % of total energy requirements based on the AMDR for the fat of 15–30 %^(16)^. Table 4 summarises the household composition and daily nutritional needs for each household member.

**Table 4.** Recommended nutrient intake reference values (daily) for each member of THFB household

Household member	Age	PAL	Energy (kcal)	Protein (g)	Fat (kcal)	Vit-A (μg)	Iron (mg)	Zinc (mg)	Calcium (mg)
Boy	2–3	1⋅45	1125	12	337⋅5	400	11⋅6	8⋅3	500
Boy	5–6	1⋅55	1475	16⋅2	442⋅5	450	12⋅6	9⋅6	600
Girl	13–14	1⋅75	2375	41	712⋅5	600	65⋅4	14⋅4	1300
Male adult	37	1⋅9	2570	46	771	600	27⋅4	17⋅1	1000
Female adult	28	1⋅9	2200	37	660	500	58⋅8	9⋅8	1000
Female adult	45–50	1⋅6	1950	35	585	500	58⋅2	9⋅8	1000
Total			11 695	187⋅2	3508⋅5	3050	234	69	5400

THFB, typical household food basket.

THFBs were designed to reflect likely household consumption of foods over time by converting per capita food consumption (kg) into daily amounts. The THFB were constrained by food expenditure and consumption data for quintile-1. A broad range of foods were selected, including seasonally consumed foods. For example, it is likely that milk is not consumed daily in quintile-1; however, it was included in the basket in small amounts relative to actual consumption over the year. Also, the inclusion of a variety of foods consumed at different times in the year, allowed us to analyse the effect of wider food price changes on the diet.

For each THFB, the total cost of the basket was calculated using food price data for the Pre- and Post-COVID-19 period, i.e. 2019 and 2020.

Using additional evidence on food consumption behaviours and coping strategies for poor households, we quantified this assumption into proportionate reduction of different food items, to nullify the effect of increased total THFB cost over the reference period. The reduction of weightage was applied uniformly to all districts.

The main computational steps are summarised as follows:

1. The total cost of the THFB using prices from our two reference points were calculated separately and a price difference (*D*) for each of these was obtained.
2. A weight (*W*) of food group expenditure reduction was established. This was 2⋅5 for fruits and dairy, 2 for vegetables, 1 for roots, tubers, pulses, legumes and nuts, 0⋅5 for cereals and grains, while sugar and oil quantities were not given a ratio, i.e. not reduced.
3. Each weight (*W*) was applied to the total cost difference (*D*), to calculate the reduction in expenditure of each food item needed, to maintain the original daily budget for food.

The weight given to each food group was estimated based on evidence of food price sensitivity in the South Asian region and changes in food consumption for the quintile-1 due to food price increases. Food group price sensitivity, from highest to lowest, were dairy, fruits, vegetables, beans/legumes, roots and tubers, then cereals and grains^(25,26)^. In our analysis, no weight was given to oil and sugar, due to the assumption that reductions in calorie rich foods would be avoided where possible.

The cost reduction for each food item was calculated based on a formula, applying the weights mentioned above. The quantity for each food item was then adjusted as per the reduced costing. Finally, the nutrient composition of the meal based on reduced food quantities was calculated using the MPT and compared with the nutrient composition of the baseline (2019) THFB.

## Results

### Food price changes

**Fig. 1.**
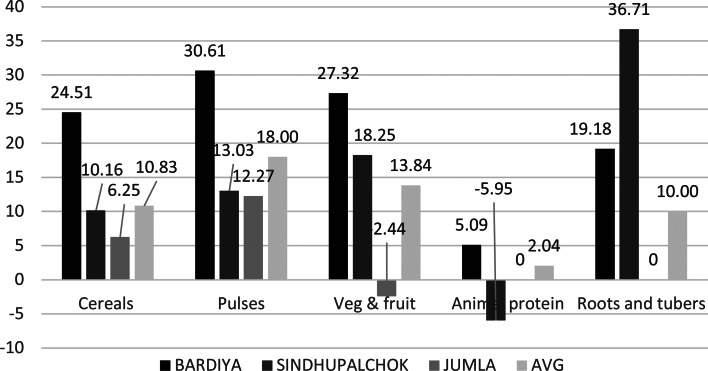
Percentage increase in food commodity prices between June 2019 (Pre-COVID-19) and May/June 2020 (Post-COVID-19) by food group by study district.

Fig. 1 indicates the percentage change in price by the food group for each district.

On average, across the three districts, data show a substantial increase in prices for almost all food groups. Pulses and vegetables and fruits show the highest average price rise at 18 and 14 %, respectively, followed by roots and tubers and cereals at around 10 %. Animal proteins show a very marginal increase in prices at 2 %.

#### Inter-district price variations by food groups

There is large inter-district variation in terms by food group. For example, in Sindhupalchok, cereal prices increased marginally by 6⋅2 %, while in Bardiya, cereal prices increased by 25 %. For fruit and vegetables, prices rose substantially in Bardiya (27 %) and Sindhupalchok (18 %), on the other hand, Jumla shows a decrease of 2⋅4 %. For roots and tubers, the inter-district price increase range is very wide, from 0 % in Jumla to 37 % in Sindhuplachok.

The food group price changes mask significant intra-food group price variations. For example, in Bardiya, in the cereals food group, price of wheat flour and rice has marginally increased, while the price of dry maize has increased from 52 Nepali Rupee (NPR)/kg to 80 NPR/kg. Similarly, in Jumla, the rise in cereal prices is primarily driven by a single commodity, i.e. *marsi rice*.

### School meals

#### Cost analysis

As can be seen in Table 5, the unit cost (child/meal) has shown a similar percentage increase in Sindhupalchok and Jumla of between 6 and 7 %, while in Bardiya, the increase is 10⋅5 %.

**Table 5.** Meal cost comparison (SMFB)

District	2019	2020	%Age change
Bardiya	16⋅34	18⋅05	10⋅47
Sindhupalchok	13⋅74	14⋅68	6⋅84
Jumla	14⋅50	15⋅44	6⋅48

SMFB, school meal food basket.

#### Diet quality analysis

A summary for the three districts is presented in Table 6. The column ‘%age change’ refers to the relative change in quantity between the two points in time, i.e. 2019 and 2020. ‘%RDA’ is used here. ‘2019 RDA’ and ‘2020 RDA’ refer to percentage RDA in a weekly context to suggest the amount of nutrient supplies in a weekly cycle as a percentage of cumulative RDA for six days for each nutrient.

**Table 6.** Comparative change in nutrient composition (SMFB)

Nutrient	Sindhupalchok			Bardiya			Jumla		
	Percentage change	2019 RDA	2020 RDA	Percentage change	2019 RDA	2020 RDA	Percentage change	2019 RDA	2020 RDA
Calcium (mg)	−8⋅72	25⋅04	22⋅86	−14⋅49	24⋅61	21⋅04	−9⋅25	24⋅38	22⋅12
Energy (kcal)	−6⋅65	20⋅66	19⋅28	−12⋅42	20⋅37	17⋅84	−11⋅82	26⋅20	23⋅10
Fat (g)	−1⋅50	7⋅98	7⋅86	−6⋅15	9⋅64	9⋅05	−2⋅72	10⋅01	9⋅74
Protein (g)	−6⋅90	52⋅83	49⋅18	15⋅19	52⋅28	44⋅34	−12⋅12	75⋅81	66⋅63
Vit-A (mcg)	−5⋅32	4⋅73	4⋅48	−11⋅80	26⋅35	23⋅24	−10⋅91	29⋅21	26⋅02
Zinc (mg)	−6⋅64	25⋅36	23⋅67	−9⋅89	23⋅47	21⋅15	−11⋅87	28⋅18	24⋅83
Iron (mg)	−7⋅47	25⋅83	23⋅91	−12⋅58	31⋅87	27⋅86	−8⋅08	65⋅90	60⋅57

SMFB, school meal food basket; RDA, recommended daily allowance.

From Table 6, while the percentage change in weekly RDA might not appear substantial in some cases, a small percentage change in RDA can reflect a significant reduction in the actual amount of nutrient supplied. For example, for Sindhupalchok, for vitamin-A, the RDA has reduced by less than one percentage point from 4⋅73 % in 2019 to 4⋅48 % in 2020, but the decrease in terms of the actual intake of vitamin-A is 5 %.

**Fig. 2.**
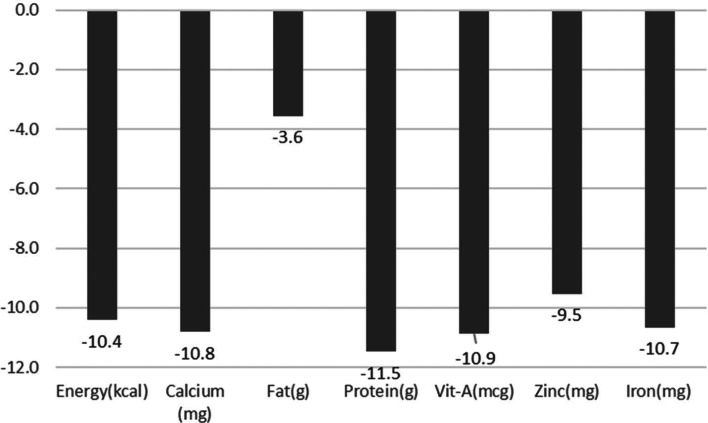
Average change (%) in nutrient quantities across study districts for school meal food basket between June 2019 (Pre-COVID-19) and May/June 2020 (Post-COVID-19), due to food price inflation over the reference period. Units used: mg – milligram, mcg – microgram, g – gram and kcal – kilocalorie.

Overall, nutrient intake is negatively impacted across all nutrients in the three districts as can be seen from Fig. 2. On average, protein shows the highest decline at 11⋅5 %, followed by energy at over 10 %. All micronutrients show large declines ranging from 9⋅5 % for zinc to 11 % for vitamin-A.

**Fig. 3.**
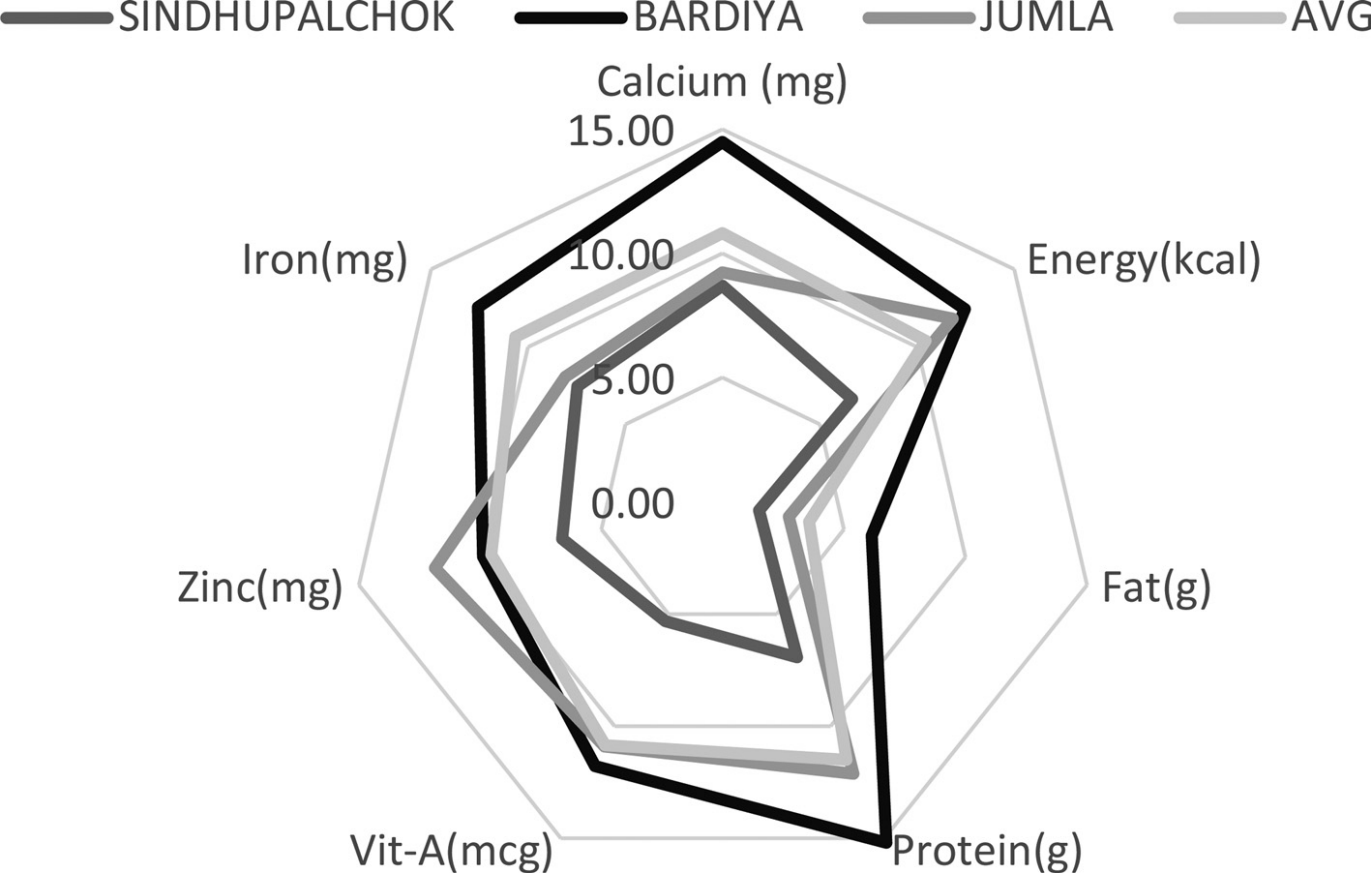
Percentage decrease in key nutrients by study district and average, for school meals food basket between June 2019 (Pre-COVID-19) and May/June 2020 (Post-COVID-19). Scale (0⋅00–15⋅00) represents the magnitude of decrease. Units used: mg – milligram, mcg – microgram, g – gram and kcal – kilocalorie.

Fig. 3 illustrates the relative changes for seven nutrients for each of the study districts. The decrease in nutrient levels during the reference period is the largest and substantially above average for Bardiya for all nutrients, except zinc. In Sindhupalchok, the decrease in nutrient levels during the reference period is the least. Jumla is close to the average for almost all nutrients. As can be observed from the figure, the general pattern in a change in nutrient levels, proportionate to each other, is quite similar for all three districts.

We finally calculated the effect of a unit increase in cost, i.e. 1 Nepalese rupee (1 NPR = 0⋅008 USD) increase on the level of nutrient intake, from our baseline cost of 2019, findings are summarised in Table 7. The impact of 1 NPR cost increase is largest in Jumla, for most nutrients. It is useful to look at the range across districts, as representing the potential impact of a 1 rupee increase on diet quality in Nepal. A 1 NPR increase in SMFB cost results in a 5⋅66–12⋅63 % decrease in micronutrients and a 7–12⋅57 % decrease in energy content, across districts.

**Table 7.** Percentage effect on nutrients for 1 NPR increase in meal cost by district (SMFB)

Nutrient	Sindhupalchok	Bardiya	Jumla
Calcium (mg)	−9⋅28	−8⋅47	−9⋅84
Energy (kcal)	−7⋅07	−7⋅26	−12⋅57
Fat (g)	−1⋅60	−3⋅59	−2⋅89
Protein (g)	−7⋅34	−8⋅88	−12⋅89
Vit-A (mcg)	−5⋅66	−6⋅90	−11⋅61
Zinc (mg)	−7⋅06	−5⋅78	−12⋅63
Iron (mg)	−7⋅95	−7⋅36	−8⋅59

SMFB, school meal food basket.

### Typical household food basket

#### Cost analysis

Table 8 summarises the total cost of food per day and the percentage increase over the reference period, for a household. The increase in cost varies from 7 % in Jumla to around 11 % in Bardiya and Sindhupalchok.

**Table 8.** Cost comparison of THFB by district

District	2019	2020	%Age change
Bardiya	296⋅9	330⋅2	11⋅2
Sindhupalchok	266⋅0	295⋅0	10⋅9
Jumla	205⋅5	219⋅9	7

THFB, typical household food basket.

#### Diet quality analysis

The results for the three districts are summarised in Table 9. Column ‘%age change’ refers to the relative change in quantity between the two time points. ‘2019 RDA’ and ‘2020 RDA’ refer to percentage RDA to indicate the amount of nutrient supplied per day as a percentage of cumulative RDA for six days for each nutrient.

**Table 9.** Comparative change in nutrient composition (THFB)

Nutrient	Sindhupalchok			Bardiya			Jumla		
	2019 RDA (%)	2020 RDA (%)	Percentage change	2019 RDA (%)	2020 RDA (%)	Percentage change	2019 RDA (%)	2020 RDA (%)	Percentage change
Calcium (mg)	69	58	−17	40	28	−35	40	32	−23
Energy (kcal)	101	101	0	107	100	−8	80	75	−9
Fat (g)	48	46	−4	51	49	−7	25	21	−13
Protein (g)	159	148	−7	169	155	−10	142	126	−16
Vit-A (mcg)	25	18	−27	86	55	−44	13	7	−69
Zinc (mg)	108	98	−9	89	83	−8	78	72	−11
Iron (mg)	47	41	−14	46	38	−19	41	32	−32

THFB, typical household food basket; RDA, recommended daily allowance.

While percentage change shows the actual reduction in intake over the reference period, changes in the proportion of the RDA targets that were met by the household for some nutrients are also quite substantial. For example, in Bardiya, for vitamin-A, the intake as a percentage of household RDA has decreased from 86 to 55 %. Similarly, in Jumla, iron shows almost a 10 % decline in the household intake. While looking at the different districts, it is important to note from the figures in ‘2019 RDA’ that there is considerable variation in the baseline diet quality of the THFBs. A comparatively higher percentage decrease for some nutrients over the reference period is simply indicative of the greater impact of food inflation on the particular THFB and not its actual diet quality.

**Fig. 4.**
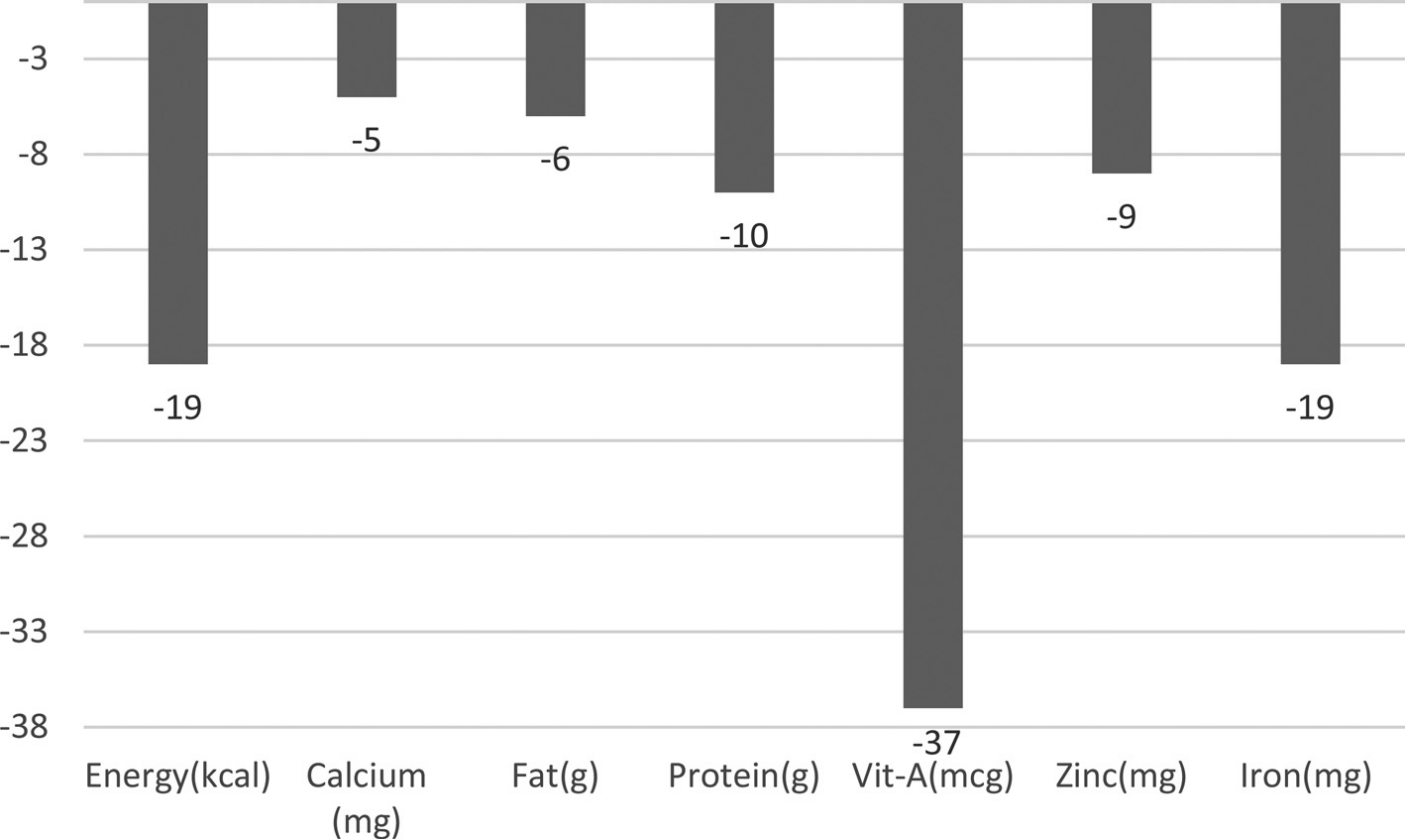
Average change (%) in nutrient quantities across study districts for typical household food basket between June 2019 (Pre-COVID-19) and May/June 2020 (Post-COVID-19), due to food price inflation over the reference period. Units used: mg – milligram, mcg – microgram, g – gram and kcal – kilocalorie.

Fig. 4 illustrates the average changes in nutrient levels across districts. In terms of macronutrients, the average reduction is relatively lower for energy and fat, compared to other macronutrients. This is partly a function of weighted reduction methods, used for THFB meal adjustment, which was lower for energy-dense foods. For micronutrients, vitamin-A content reduces by the largest average amount at 37 % followed by iron at 19 %. Reduction in zinc is quite low due to the high zinc content in whole grain cereals in all the THFBs.

**Fig. 5.**
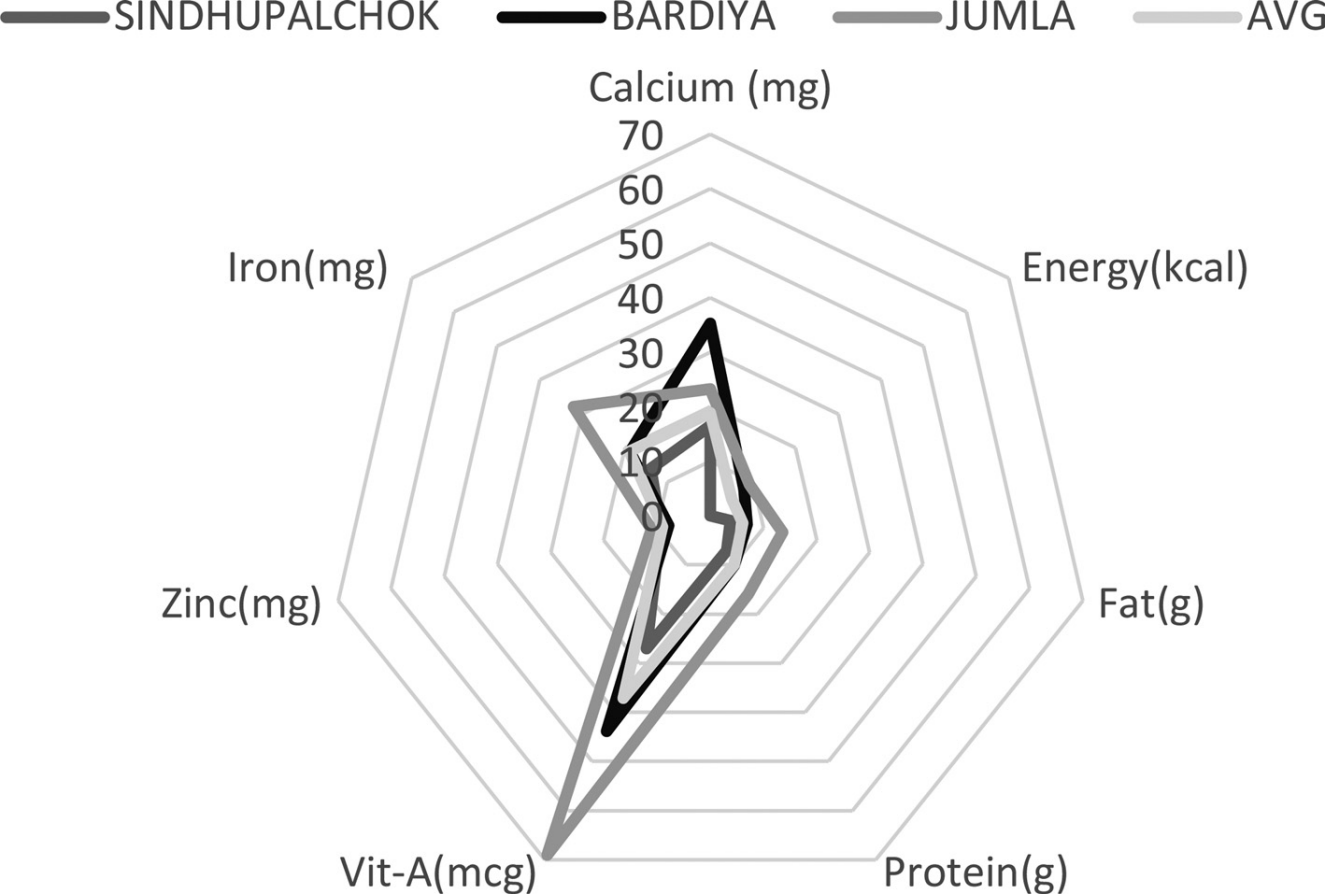
Percentage decrease in key nutrients by study district and average, for typical household food basket between June 2019 (Pre-COVID-19) and May/June 2020 (Post-COVID-19). Scale (0⋅00–15⋅00) represents the magnitude of decrease. Units used: mg – milligram, mcg – microgram, g – gram and kcal – kilocalorie.

Fig. 5 illustrates the relative changes for the seven nutrients for each of the study districts. Sindhupalchok and Jumla follow a similar pattern, although the magnitude of reduction in Jumla is substantially greater than the other two districts. Overall, Sindhupalchok shows the least reduction for all nutrients and is below average. Reductions in vitamin-A, iron and calcium appear most prominent for all districts.

Finally, we calculated the changes in nutrient intake (%) when a unit increase in cost, i.e. 10 NPR, is applied to the baseline cost of 2019. Findings are summarised in Table 10. We chose 10 NPR as the unit for the THFBs, compared to 1 NPR for SMFBs to reflect the much larger budget scale of the THFBs. For every 10 NPR increase in the cost of THFB, vitamin-A is reduced by 9–48 %, iron by 5–22 % and zinc by 3–8 %. The wide range represents substantial inter-district variation, Sindhupalchok showing substantially lower percentage reduction for all nutrients compared to the other two districts.

**Table 10.** Percentage change of nutrients for 10 NPR increase in meal cost by district (THFB)

Nutrient	Sindhupalchok	Bardiya	Jumla
Calcium (mg)	−5⋅72	−31⋅54	−16⋅14
Energy (kcal)	0⋅00	−7⋅07	−6⋅41
Fat (g)	−1⋅30	−6⋅14	−9⋅33
Protein (g)	−2⋅44	−9⋅02	−11⋅05
Vit-A (mcg)	−9⋅34	−39⋅10	−47⋅79
Zinc (mg)	−3⋅23	−6⋅96	−7⋅65
Iron (mg)	−4⋅83	−16⋅82	−22⋅18

THFB, typical household food basket.

## Discussion

The present study shows that food prices have increased substantially over the reference period across all food groups except animal proteins. The findings highlight substantial differences in price changes between the study districts. For example, in Bardiya, prices for fruits and vegetables have increased by 27 %, while in Jumla, the prices decreased by 2⋅4 %. The inter-district difference in price inflation can be primarily attributed to the nature of agriculture markets and trade, with nationally and internationally integrated markets being more vulnerable to disruptions in transport. For example, Bardiya district is well integrated in the national agriculture supply chain, due to its location and topography. The closest major agriculture market is in Nepalgunj which is around 2 h by road. By comparison, Jumla district is located in the high mountain ranges of the Himalayas with an average elevation of 2250 m. The closest major agricultural commodity market is in Surkhet which is around 11 h travel by road. Agriculture markets in Jumla, especially for perishables like vegetables and fruits are mostly localised, and people depend on their own production for household consumption.

As expected, increased food prices have resulted in a cost increase for SMFB and THFB. For SMFB, the percentage increase in Sindhupalchok and Jumla is between 6 and 7 %, while in Bardiya, the increase is 10⋅5 %. For THFB, the increase in cost varies from 7 % (450 NPR/month) in Jumla to around 11 % (1000 NPR/month) in Bardiya and Sindhupalchok. This increase of 450 and 1000 NPR represents around 6 and 13 % of the monthly household food budget and around 4 and 8 % of the overall HH consumption, respectively (authors calculation based on DHS 2016).

The estimations based on two different approaches developed in the present paper, i.e. SMFB and THFB, are representative of two very distinct dietary strategies. The SMFBs-based nutrient analysis provides estimates for nutritionally balanced and diverse diets and THFB-based analysis provides estimates for typical diets of poor households. The findings based on both approaches clearly suggest that even a nominal increase in food basket cost can have marked negative implications on the diet quality of the meals, impacting the intake of both macro and micronutrients. A 1 NPR increase in the unit cost of SMFB, decreases micronutrient intake by 5⋅66–12⋅63 % and energy by 7–13 %. A 10 NPR increase in the unit cost of THFB decreases micronutrient intake by 9–69 % and energy by 0–9 %. While there are noticeable inter-district variations in the scale of impact, and between SMFB and THFB, the overall conclusion remains consistent.

The significant reduction in the intake of some micronutrients is of particular concern given the severity of the current deficiencies in zinc, iron and vitamin-A, as highlighted in the national micronutrient survey^(21)^. Deficiencies in these micronutrients constitute a serious public health crisis and can lead to poor growth, lower school performance, a wide range of diseases, ailments, increased morbidity and mortality^(27–29)^.

The change in consumption patterns to cope with increased prices will have the most serious impact on the diet quality of poor households. In terms of intra-household food allocation, women and children are likely to suffer disproportionately^(8)^. This is especially detrimental during pregnancy, where even small changes in the micronutrient content of diets are associated with significant differences in fetal and infant growth^(3)^.

The analysis of the present paper combined with the evidence from other studies suggests that COVID-19 control measures are potentially contributing to undermining food and nutrition security, with the poorest being hit the hardest and young children potentially facing lifelong consequences. The evidence emphasises the urgent need to address the impending food security-related public health crisis by implementing mitigating and remedial strategies. As schools continue to remain closed, weekly take-home rations should be explored with clear safety protocols. The school feeding platform can also be leveraged as a public distribution system by providing extra rations during this crisis. National food supply reserve agencies like the Food Management and Trading Company of Nepal can also play a vital role in providing support to food markets and supplies such as ensuring procurement after harvest and enabling aggregation.

The findings of the present study also indicate the importance of resilient localised food systems. This is not to suggest that food supply chains should not be optimally integrated but where possible, local production systems need to be strengthened through deliberate input and output support interventions. Finally, the scale of the current crisis is an emphatic reminder of the need to have an integrated public health-food system approach in response to such a crisis. This can include rapid public procurement of agriculture commodities, distribution of micronutrient supplements and fortified foods, and targeted antenatal care.

The present study looks at the situation two months post-implementation of control measures. As the public health crisis is still unfolding, over the next few months, food prices can be volatile depending on issues of storage, international prices, disruption to summer planting season, etc. It will be useful to conduct multiple small rapid surveys on food production and consumption over the next few months to capture the diverse effects of the pandemic on food and nutrition security on a continuing basis. This can provide useful evidence to inform decision-making on contextually relevant food security interventions.

Some major limitations of the present study need to be highlighted. First, seasonality is an important element in food production and consumption which could not be included in the research design. Second, the research design assumes that purchasing power is constant over our reference period. Due to reduced incomes and wages, the actual impact on diet quality, in all probability is more substantial than indicated by the findings and estimations of the present paper. Notwithstanding the limitations, the present paper provides useful insights on the magnitude and nature of the potential effects of COVID-19 pandemic on food prices and nutrition security. It also highlights the very critical and immediate need for sustained food and nutrition support as a priority, to ensure that the hard-won public health gains of recent years are not entirely lost.
